# Attending to Gyn‐Ecology: Managing the ‘Mess’ of Recurrent Vulvovaginal Thrush

**DOI:** 10.1111/1467-9566.70121

**Published:** 2025-12-19

**Authors:** Tori Ford, Sue Ziebland, Sarah Tonkin‐Crine, Gail Hayward, Abigail McNiven

**Affiliations:** ^1^ Nuffield Department of Primary Care Health Sciences University of Oxford Oxford UK

## Abstract

Previous social science research on gendered experiences of thrush and vulvovaginal health has overlooked, or inadvertently sanitised, the materiality of having a body. Therefore, this study aims to return attention to the material, sensorial, and corporeal dimensions of living with recurrent thrush and use this to broaden our understandings of gendered embodiment. We present findings from a qualitative study of interviews with 32 people including women, trans, nonbinary and gender diverse people. This paper examines the bodily experiences of recurrent vulvovaginal thrush through a feminist materiality lens, drawing on concepts of ‘leaky bodies’ and microbiological relationships with the ‘not‐me’/‘not‐not‐me’. We present the multidimensional experiences of managing recurrent vulvovaginal thrush, examining the ‘mess’ it creates, the vigilance required to monitor microbial imbalances, the negotiation of gendered perceptions of ‘grossness’ and the compromises individuals make between physical and emotional dis/comfort. Through attending to the material, microbial, and gendered imaginaries and realities involved in managing the ‘mess’ of recurrent thrush, we explore the additional considerations and challenges experienced by those with lived experience currently missing from research.

## 1 Introduction

Recurrent vulvovaginal candidiasis, known colloquially as recurrent thrush, involves repetitive fungal infections resulting from yeast overgrowth in the vaginal microbiome (Denning et al. 2018). Symptoms include genital itching, burning, painful intercourse and abnormal vaginal discharge known unflatteringly as resembling ‘cottage cheese’ (Saxon et al. 2020). Roughly 75% of people with vaginas will experience thrush at least once, with symptoms that are acute, transient and easily managed with pharmacy care. However, for 6% of cases (an estimated 1.2 million women in the UK), thrush becomes a repetitive condition, with four or more episodes a year (Denning et al. 2018). Management is typically delivered in primary care using long‐term courses of antifungal medication. However, management challenges remain due to ambiguous symptoms, limitations with existing diagnostic tools and a lack of continuity of care (Ford et al. 2024). Past research studies haveidentified that thrush is a condition associated with shame, stigma and embarrassment (Ford et al. 2024; Strydom et al. 2022; Chapple et al. 2000; Chapple 2001); however, few have detailed how these feelings intersect with the material, microbial, and gendered realities of living with recurrent thrush. These linkages remain unspoken in the literature.

This paper returns attention to how people experiencing recurrent thrush embody unruly and uncomfortable bodies that produce secretions, residues, sensations and spillages. We examine the bodily experiences of recurrent vulvovaginal thrush through a feminist materiality lens, drawing on concepts of ‘leaky bodies’ and microbiological relationships with the ‘not‐me’/‘not‐not‐me’ (Longhurst 2000; Grosz 1994; Langdridge and Flowers 2013).

We present how participants attend to ‘cleaning up’ the material, microbial, and gendered ‘messes’ that recurrent thrush and its attempted management cause.

In doing so, we contribute to the growing field of feminist health studies by foregrounding the lived experiences of recurrent thrush, challenging the sanitisation of its material realities and providing insight into how individuals weigh the needs of their body, microbiome and self.

### 1.1 Existing Gendered Narratives Around Vulval Discomfort

Previous social science research on gendered experiences of thrush and vulvovaginal health has overlooked, or (perhaps inadvertently) sanitised, the materiality of having a body.

This is perhaps due to foregrounding other aspects of experiences (such as the psychological), or influences of ‘academic squeamishness’ referring to the research, publishing and funding norms of avoiding aspects of topics seen as disgusting, repulsive or otherwise inappropriate for scholarly consideration (Longhurst 2000; Longhurst and Johnston 2014).

Previous research studies have focused on social impacts of recurrent thrush, including emotional, mental and psychological impacts (Ford et al. 2024; Fukazawa et al. 2019). Existing studies that examine thrush through a gendered lens centre on cis‐women’ experiences. Chapple (2001) found that British women with thrush felt a sense of ‘shame’ and ‘spoiled identity’ because thrush was not immediately visible, but something that they saw as being ‘discreditable’. An Australian interview study found that people's self‐esteem and confidence were impacted by recurrent thrush due to embarrassment and shame (Strydom et al. 2022). Adolfsson et al. (2017) highlighted how Swedish participants with recurrent thrush felt ‘undesirable as women’.

Wider literature on vulvovaginal conditions that produce similar symptoms to thrush, for example, itching, burning and discomfort, are also located within similar frameworks. Vulvodynia, which causes persistent or recurrent pain, discomfort and burning of the vulva has been highlighted as resulting in perceptions of ‘failed’ or ‘threatened’ femininity (Kaler 2006; Marriott and Thompson 2008). Experiences of feeling ‘defeminised’ have been reported in relation to vulvodynia (Ayling and Ussher 2008) and lichen sclerosus, a chronic skin condition affecting the vulva. Research into experiences of recurrent thrush typically aligns with these larger gendered narratives around ongoing vulvovaginal discomfort. While the emotional and psychological discomforts are presumably premised on the material, corporeal and sensorial realities, often these are backgrounded and left unspoken in the context of research on experiences of vulvovaginal conditions including thrush.

Further, this approach can present an oversimplified understanding of gender. Research studies have not yet included the voices of gender‐diverse, nonbinary or trans people with recurrent vulvovaginal thrush who may disrupt these prevalent narratives.

### 1.2 Bodily Fluids, Filth and Femininity

Attempts to reckon with ‘academic squeamishness’ have been explored through literature which foregrounds physical bodies and materiality (Longhurst and Johnston 2014). The concept of the ‘leaky body’ has been described by Grosz (1994) regarding how female bodies have been socially constructed as ‘leaking, uncontrollable, seeping liquid; as formless flow; as viscosity, entrapping, secreting’. This idea of the ‘leaky female body’ contrasts female embodiment as defiling boundaries unlike the (white, young, non‐disabled, cis‐gender) male body that is positioned as regulated, contained and in control (Longhurst 2000; Jenkins et al. 2018; Shildrick 1997). Bodily fluids and fleshy bodies have often been left out of research and writing for being ‘too banal, too material, too feminist, too mysterious, too Other’ (Longhurst and Johnston 2014).

The aversion to bodily fluids is rooted in multifaceted social and cultural understandings of filth, purity and pollution. As Lupton (1996) described, ‘bodily fluids threaten to engulf, to defile; they are difficult to be rid of, they seep and infiltrate’. Previous ways to understand leaky bodies have been through the lenses of disgust and abjection. Douglas (1966) first articulated ‘dirt’ as matter out‐of‐place which speaks to the bodily, emotional and social dimensions of illness. Kristeva (1982) defined the abject as that which ‘disturbs identity, system and order. What does not respect boundaries, positions, rules. The in‐between, the ambiguous, the composite’.

Previous work on leaky bodies has included exploration of how people manage bodily fluids such as menstrual blood (Bobel 2010), breast milk (van Amsterdam 2015), urine (Jordan 2007), faeces (Priddis 2015) and those associated with birth (Lupton and Schmied 2013). Recurrent thrush and its associated vaginal discharge share features with other forms of leakages, yet also present distinct considerations. Thrush‐related discharge may be less visible to others than, for example, a menstrual leak or urinary incontinence, which may afford greater ease of concealment, but also obscure the ability to tend to the discomfort. Across these examples, concealment and secrecy continue to be central, with individuals expected to manage leaks through appropriate ‘tools’ (period products, incontinence pads or antifungal medication) and constant vigilance around timing, detection and ‘catching’ leaks early. For some, these timings may become cyclical or repetitive; for others, they remain irregular and unpredictable. Discourses around these leaks are shifting: menstruation has become increasingly open to public discussion, whereas incontinence is moving away from being normalised (with age or childbirth) and being reframed as a condition worthy of medical attention. Vaginal discharge, by contrast, has only recently begun to attract attention in efforts toward destigmatisation, and remains more marginal in public and clinical discourse. Despite its prevalence, vaginal discharge and thrush have received comparatively little sustained scholarly attention as a secretion in its own right.

Vaginal discharge has been seen as particularly polluting, due to associations of femininity, cleanliness and containment. Karasz and Anderson (2003) showed that people with ‘healthy’ vaginas often see their genitals as ‘unclean’ or ‘disgusting’ with all vaginal discharge being highly stigmatised. Therefore, vaginal secretions, fluids and flows are particularly met with these visceral reactions, especially when associated with infection or disease. Female bodies have previously been framed as mysterious, dirty and naturally prone to infection and disease (Braun and Wilkinson 2001), with the dark and wet vagina being seen as the ‘ideal breeding ground’ for yeast (Overend 2011).

Stereotypes of vaginas as inherently ‘unclean’ also have material consequences. People who embody ‘leaking’ are socially expected to monitor and regulate their bodies (Shildrick 1997). There is a billion‐dollar ‘feminine‐hygiene’ industry which promotes ‘body work’ such as deodorising and grooming vulvas and vaginas to be ‘attractive’, ‘clean’ and ‘fresh’, with soaps, sprays, wipes and douches (Crann et al. 2017; Jenkins et al. 2018). This language around ‘hygiene’ implies that there is dirt to clean up, whether that be blood, discharge or smell. Further, there is a tension here between presentation, containment and comportment, which do not always align with the reality of having a leaky body.

These attitudes and some practices around promoting ‘healthy’ and ‘clean’ vaginas can pose risks for thrush. For example, deodorising and douching products have been documented to disrupt the vaginal microbiome and potentially lead to infection, establishing a vicious cycle (Crann et al. 2017). Alongside ‘feminine‐hygiene’ products, the same brands often supply treatment for thrush, identifying it as another form of dirt to clean up, while also profiting from this cycle or causing other complications.

The concept of leaky bodies effectively highlights the social and cultural implications of bodily fluids and boundaries but can overlook sensorial dimensions, such as itching, burning, and irritation. Leder's (1990) framework of ‘dys/appearance’ can expand the discourse of leaky bodies beyond fluid boundaries, offering a means to theorise how discomfort itself functions as a form of leakage. Leder (1990) explains how in everyday terms, the body tends to ‘disappear’, yet in illness, sensations such as pain or itch ‘dys/appear’ making the body obtrusively present and demanding attention. These sensations can be understood as a ‘leak’ of (in)attention, that reinforces bodily awareness and distress and often compels an action (such as scratching) (Leder 1990). Whereas fluid leakages challenge the boundary between inside and outside, sensation like itch disrupts the very relationship between embodiment and attention, dragging attention back to the affected body parts and their ‘dys/appearance’ (Leder 1990). With recurrent thrush, this discomfort is not only bodily but also shaped by social discourses around femininity and pathology.

### 1.3 Microbiological Imaginaries

The microbiome, an intricate ecosystem of bacteria, fungi and viruses, has become a focal point in contemporary biomedical and sociological thought. As a site of both balance and disruption, the microbiome challenges traditional notions of bodily boundaries, often blurring the lines between self and other. Within this framework, the concept of the ‘not‐me’ has been used to explore how individuals experience the microbiological inhabitants of their bodies as both integral to and alien from the self (Langdridge and Flowers 2013). This tension between embodiment and alienation is particularly salient in conditions where microbial imbalances manifest as illness or discomfort, such as irritable bowel syndrome, HIV or autoimmune disorders. Recent studies, reveal how the microbiome can alternately be experienced as ‘me’, ‘not‐me’, or ‘not‐not‐me’ (Cohen 2017; Laursen et al. 2022). However, a significant gap remains in understanding how people relate to their vaginal microbiome, especially when yeast, a natural inhabitant of the vaginal microbiome, overgrows and becomes pathogenic. An exploration of recurrent vulvovaginal thrush, as yeast which grows (and overgrows) in the microbiome and how people contend with its existence or attempted eradication is missing.

## 2 Methods

This paper presents a qualitative study of interviews with 32 individuals with recurrent vulvovaginal thrush. Interviews were conducted between May 2022 and June 2023. Interviews were conducted online using video calls (27) on the telephone (6) or in‐person (1), based on participant preference. Two individuals initially consented to participate but later withdrew; in line with ethical protocol, no data from these individuals were included in the analysis.

The research team holds diverse expertise within qualitative health, social science, clinical expertise and lived experience. A patient representative group was assembled to guide this research, aiding with study design, recruitment, topic guides and disseminations. Interviews were all conducted by a doctoral qualitative researcher who is a cis‐gendered white woman. The interviewer disclosed to participants having lived experience of recurrent vulvovaginal thrush. This positionality influenced interview dynamics as some participants reported feeling more comfortable due to shared experience, but also carried risk of participants assuming the interviewer had prior knowledge, leading to less elaboration. To address these dynamics, the interviewer used a range of prompts and remained reflexive using research journaling and team discussions.

Recruitment began with a patient representative group meeting to create and disseminate patient‐facing materials that were circulated through various online and in‐person channels. These locations included GP surgeries, sexual health clinics, pharmacies and community centres across England. We also circulated study information online through a dedicated webpage, newsletters, online support groups for vulval health conditions, and social media. We also hosted an online event with Medical Herstory on ‘Storytelling to Undo Stigma: Vulvovaginal Health’ and a Facebook Live with the Vulval Pain Society.

We used a maximum variation sampling approach to ensure diversity in ethnicity, socioeconomic status, gender, sexuality and age (Meyer and Xyländer 2018). Sampling involved looking for both demographic variation and a range of experiences. We took a considered approach to recruitment, prioritising those from seldom heard groups and reaching out to potential participants to ask about experiences we saw missing from our sample (such as experiences of pregnancy, and those who had success with long‐term medications).

The sample included 32 people. 28 people identified as cis‐women, two as nonbinary, one as trans and one as gender fluid. The sample included a range of ethnic minority backgrounds and sexual orientations. Ages ranged from 21 to 60 years‐old. Twenty‐nine people described their thrush as recurrent, cyclical or repetitive, and three labelled it as persistent or chronic. Participants reported having recurrent thrush for a range of durations from a few months to over a decade. See Table 1 for further details.

**TABLE 1:** Self‐described characteristics of patient sample.

Name	Age	Gender	Sexuality	Ethnicity
KJ	42	Gender fluid	Heterosexual	White
Ayesha	25	Woman	Heterosexual	Pakistani
Teddy	21	Non‐binary	Lesbian	White
Nancy	37	Woman	Heterosexual	White
Emily	32	Woman	Heterosexual	White
Aditi	22	Woman	Heterosexual	Indian
Sai	24	Woman	Heterosexual	Indian
Etta	50	Non‐binary	Bisexual	White
Ruby	40	Woman	Heterosexual	Black British
Beth	25	Woman	Bisexual	White
Laura	42	Woman	Heterosexual	White
Imani	35	Woman	Heterosexual	Black
Jody	26	Woman	Bisexual	White
Elliott	30	Trans	Queer	White
Billie	25	Woman	Heterosexual	White
Joy	43	Woman	Heterosexual	White
Sasha	34	Woman	Heterosexual	Black
Zoya	33	Woman	Heterosexual	Pakistani
Kayla	42	Woman	Lesbian	White
Lydia	26	Woman	Heterosexual	White
Leah	26	Woman	Heterosexual	White
Anna	34	Woman	Bisexual	Mixed race
Georgia	27	Woman	Heterosexual	White
Emma	41	Woman	Heterosexual	White
Francine	60	Woman	Heterosexual	White
Harry	25	Woman	Heterosexual	White
Julia	36	Woman	Heterosexual	White
Hannah	31	Woman	Heterosexual	White
Chloe	30	Woman	Heterosexual	White
Imogen	29	Woman	Bi‐romantic	White
Rowan	24	Woman	Bisexual	White
Sarah	35	Woman	Heterosexual	White

The study methods were approved by the NRES Committee South Central—Berkshire REC reference (12/SC/0495HTO). Informed consent was discussed before interviews. The interviews began with a narrative section where participants were invited to say what had happened since they first started to suspect there was something wrong (Grob et al. 2016). They were encouraged to speak openly and without interruption to capture the aspects that were important to them. Then, a semi‐structured interview guide was used to direct the conversation and expand on areas that participants raised in the narrative section of the interview (Ziebland 2013). This topic guide was designed with patient representatives. Participants were reminded that they did not have to respond to any questions that they were not comfortable answering. Interviews lasted between 45 min and 2.5 h (over two sessions).

Interviews were recorded and transcribed verbatim, then anonymised and uploaded to NVivo12. Review of transcripts or a summary were offered to participants, 1 person requested a transcript and the rest opted for a summary. The first author carried out interviews, reflexive thematic analysis, and initial theme generation which were then reviewed in discussion with the wider study team. All codes relevant to bodily experiences and gender were then analysed using the one sheet of paper (OSOP) mind mapping method (Ziebland and McPherson 2006). This approach involves taking a category (in this case embodied experiences) and capturing the full range of issues raised by individual respondents, allowing the analyst to see what was common, what was unusual and ensure that no respondent's contribution is overlooked (Ziebland and McPherson 2006).

Further analyses and extracts from the interviews were used to develop a public‐facing online resource to support patients and their healthcare professionals (https://hexi.ox.ac.uk/Recurrent‐Vulvovaginal‐Thrush). Participants were informed about this output in the participant information sheet; it was discussed during informed consent, and after interviews, participants chose how they would like to be represented on the website (through video, audio, animation and/or text clips).

## 3 Findings

A number of prominent themes were identified in the data. Themes relating to embodiment and gender were identified and analysed further as an area of interest.

Below we present the multidimensional experiences of managing recurrent vulvovaginal thrush, examining its material, microbial, and gendered considerations including: handling physical and sensorial mess, monitoring the vulvovaginal microbiome, negotiating gendered perceptions of ‘grossness’ and making compromises between physical and emotional dis/comfort. Participants chose the pseudonyms or first names that are used throughout this paper.

### 3.1 Handling Material and Sensorial Mess

Although vaginal discharge was often a sign of a healthy self‐cleaning vagina, participants described thrush as different. Differences were noted in quantity, texture, colour and odour. Sasha said ‘*the discharge was just not the way it usually is, it was more like cottage‐cheese*’. Others said the discharge was ‘curdy’ or ‘creamy’. Zoya described the texture of thrush pieces as ‘*it's really different, it's little bits of discharge*’. Imogen added it is ‘*thicker, whiter*’. The amount of discharge was also noted, as Sai explained ‘*my panties used to get properly drenched*’. Thrush discharge was often described as odourless, but others said it could be ‘yeasty’ and Zoya said it has *‘a really distinctive smell’.*

In an attempt to ‘clean up’ thrush discharge, participants changed their underwear more frequently or attempted to hide it in their washing. KJ carried extra pairs of underwear every day in her bag. Ayesha explained spending years worried about concealing her discharge:

I'm quite conscious of when I do my washing just making sure it’s all very hidden because I know that in all my underwear you can see that discharge, and it’s been like that for years.

Participants said that while they could wipe away discharge or conceal it, they knew it could always return.

The sensations caused by recurrent thrush were described as viscerally uncomfortable Sasha described an ‘*itchy burning sensation, it's just raging, raging, raw’.* Georgia said her genitals were ‘*itchy on the inside, really burning, very painful, and really difficult to relieve’.* Teddy said: ‘*I wanted to claw my insides out, which is a really gross image’.*

Itching could sometimes cause participants to scratch, potentially bringing them into contact with blood, scabs, weeping wounds and pus. Leah explained: ‘*It's just constant really, itch, itch, itch, and it makes it bleed, then 2 weeks later I'll get it again*’. Aditi detailed the cycle of itching and scratching caused by recurrent thrush:

It was painful because maybe this is weird but like the little blob it would turn red and very … very soft and very painful just in general, overall having to itch all over that area was painful.

While scratching could provide short‐term relief from itch, it also often also led to further stinging, irritation and discomfort. Beth said that ‘*with the constant scratching, I've got lots of abrasions down there*’. Some people spoke about how they used to scratch, but over time found other ways to relieve itch as Ruby explained:

I was having to go into the bathroom and get the shower head, turn it up as hot as I could just to clear [the discharge that] was inside my vagina … I'd blast it to try and get clear it and it probably sounds weird, but I found it quite soothing because all I wanted to do was itch, which you're not supposed to do.

Visual signs of irritation were also described by participants. Emma said ‘*It's uncomfortable because the skin is a lot more delicate, it's a lot redder*’. KJ described ‘*red raw, split skin’.* Etta felt ‘*little tears, kind of stinging*’. Etta said her vulva was ‘*really inflamed*’. People described how recurrent infections made it difficult for the skin to heal between episodes.

Participants further spoke about ‘soreness’ and feeling ‘tender’. Kayla described the sensation as ‘*a constant toothache in your vagina area*’. Anna said ‘*it just felt, or feels, so dry and sore*’. The tension here between both ‘felt’ and ‘feeling’ speaks to the ways in which recurrent thrush would repeatedly appear throughout people's lives.

Trying to ‘clean up’ recurrent thrush could itself be messy. Treatment options such as pessaries were described as ‘leaking’ or ‘falling out’. Using antifungal creams and emollients could simultaneously offer relief and generate material mess. Julia said that pessaries: ‘*fall out of you over like a day period and make an absolute mess*’. Lydia similarly recalled that after putting pessaries in at night, the next day she felt ‘*the remnants of the pessary, the rest of it coming out*’. Joy found it hard to insert pessaries: ‘*I couldn't even put the pessary in, because sometimes it can be that red and swollen*’.

The presence, sensation, and appearance of discharge, itch and irritation led people to ‘clean up’ both during and in between episodes of recurrent thrush. Discharge and itch were often understood as the materialisation of an unbalanced vaginal microbiome and yeast as a microscopic material that required managing.

### 3.2 Monitoring the Vulvovaginal Microbiome

To manage or prevent recurrent thrush, participants described self‐monitoring their bodies and microbiomes. Participants described multiple ways that they understood and imagined their vagina and its microbiome, which made it a site for potential symptoms to be monitored. This process often began with trying to modify a few daily habits but might then move onto detailed routines and adjustments over time.

The idea of the vagina as a ‘breeding ground’ for yeast and bacteria was shared by participants. Etta said that ‘*we know that moulds, yeasts, whatever, like warm, sweaty, dark, you know all that*’. Anna said ‘*the doctor was like:* *your pH balance in your vagina is off, it's like a breeding ground*’.

The language used around microbiomes sometimes conceptualised recurrent thrush as an attacking invader to be anticipated, located and removed, whereas others saw it as constantly present and requiring care and balance.

Some participants articulated a struggle for control over their bodies. However, this struggle was not framed as one active agent fighting to eradicate a passive infection, but as an active negotiation between two agents: their selves and their vaginal microbiome. This relationship was sometimes framed as antagonistic, with a personified ‘angry’ vagina. Militaristic language of being ‘at war’ with one's vagina was used by some participants to describe their frustration. Teddy said ‘*it's like my vagina was just out to get me*’. Anna said that she used to hold similar views, but over time she believed that her ‘body is not my enemy’ and thrush was her vagina's way of ‘talking’ to her.

When I think about that period of time, I really think that I was like at war with my vagina, and I just wanted to have a different one; whereas I think now, when I get thrush, […] I normally go, ‘oh hang on, let me just figure out what’s going on: am I stressed? Am I eating properly? Am I tired? Have I been drinking too much?’ […] I view it more as my vagina talking to me; whereas I used to view it as my vagina trying to ruin my life.

Etta described becoming ‘friends’ with the bacteria that protect the vaginal microbiome, and understanding that there is a delicate ‘internal ecology’.

I've kind of got more of an attitude now of it’s either under control, or it’s not, and there’s a spectrum of that, but that I'm not sure it’s ever something that’s completely neutralised, and if it was, then you might actually be really poorly because we need bacteria to function, they're a big part of our internal ecology […]. So yeah, I think it’s trying to make friends with … [laughs] with the right bacteria.

Participants described needing to be ‘vigilant’ before and between recurrent thrush episodes which often required self‐monitoring not only their behaviours but the imagined materiality of their microbiomes. This involved acting in anticipation of symptoms, before any tangible, visible or sensorial signs materialised.

Recurrent thrush could re‐appear at any time and therefore participants often felt the need to be consistently self‐monitoring to predict flare‐ups. KJ said ‘*I'm a lot more vigilant*, *I'm looking out for things*’. Ruby said ‘*I'm a bit more proactive nowadays with regards to noticing what's going on*’. Etta described the ‘job’ of self‐monitoring:

It is my job to be vigilant and notice its early warning signs […] I’m much more proactive: what can I do to kind of prevent, prevent, prevent.

Over time, participants also developed carefully coordinated cleaning routines to manage their microbes. People often took care with avoiding irritating products such as ‘feminine washes’, shower gels, douches, soaps, bath bombs and scented pads, liners or wet wipes that could all trigger a flare‐up. Harry identified a ‘vicious cycle’ of potential over‐washing: ‘*I feel like I just want to clean [the vulval area], and then the more you clean, the worse it gets*’. Aditi would ‘doubly cleanse’ to avoid being ‘dirty’ but had also been told that she might be washing too often, making the skin dry and irritated. A few people were recommended emollients from their healthcare professional which they found helpful to use as a soap substitute, daily moisturiser and barrier cream, but many had not heard this advice. People also said that over time, their cleaning routines were influenced by a larger community, such as community forums offering advice.

Participants said that going to the toilet could be challenging due to increased irritation from interactions with other bodily fluids (urine, faeces, menstrual blood and sweat) and related materials (toilet paper and menstrual products such as pads and tampons). Some participants described having a specific cleaning routine after using the toilet.

After washing, routines also extended to drying off thoroughly, patting dry instead of rubbing with a towel, changing towels often, and using a hairdryer on the affected area. Some people changed their washing powder, ironed the gusset of their underwear, or modified their clothing. Participants noted that these behaviours were practiced not only while having a flare‐up but as a daily routine since having recurrent thrush.

Ayesha said that this self‐monitoring required her to be hyper‐vigilant of her body, not just while symptoms were active but in the liminal space before a flare‐up:

I always have this kind of niggling feeling in the back of my mind […]. I feel like everything has to be set in place, and I've got to have things on hand ready for a flare‐up, or I'm always thinking, ‘OK, do I feel a symptom; is it getting worse’, during those moments where I'm meant to be fully relaxed and not worrying about it.

Others spoke about particular times when flare‐ups intensified, often in relation to their menstrual cycles, pregnancy, or stressful life circumstances. Therefore, although there was a tendency to self‐monitor continuously, it was punctuated with intense self‐monitoring around these particular periods.

The pressure to self‐monitor the microbiome sometimes led participants to pre‐emptively take antifungal medication. Leah said ‘*if it's a bit yeasty, start to get that twinge, I'll take a tablet* […] *it's like when you get headaches and stuff, if you suffer with headaches you'd take paracetamol with you, wouldn't you, so it's the same sort of thing as having that safety blanket*’. Noticing the first ‘twinge’ of discomfort signalling that thrush may be imminent was seen as an important responsibility. This ‘safety blanket’ approach to having easy access to medication spoke to the unpredictability of recurrent thrush. Leah continued to say (while acknowledging that she saw this as ‘naughty’) that vigilance could also include taking medication pre‐emptively:

I do take … really naughty, I do … very occasionally I’ll take a pre‐emptive … like a just‐in‐case, if I’m going away with my husband or I know that it’s the right time and I’m just before my period, I’ll just take a capsule without symptoms.

Therefore, being vigilant required constant monitoring, being prepared and also having the right strategy or ‘equipment’ to manage as necessary and in any situations.

Managing the microbiome also involved trying to increase the presence of ‘good’ bacteria. This included participants taking probiotic supplements or making their own as Etta did ‘*It's a bit extreme, but I grow my own kefir and kombucha, which are good for positive bacteria*’. Zoya said that antibiotics had ‘*wiped out all the good stuff*’ in the vaginal microbiome and she found probiotics helpful in restoring this balance.

### 3.3 Negotiating Gendered Perceptions of ‘Grossness’

Recurrent thrush also carried psychological burdens, particularly linked to societal notions of shame, ‘grossness’, and disgust.

Participants describing the sensorial experience of recurrent thrush would often apologise for their descriptions or label them as ‘gross’, or ‘weird’. Recounting her experience, Zoya said ‘*oh, it sounds really disgusting, I don't want to describe it, but I'm going to try and give you a picture of [thrush] without grossing you out*’. Similarly, Ruby said ‘*please forgive me if I'm being too graphic*’.

Nancy said that there was a social perception around recurrent thrush and grossness, even if it was not articulated aloud.

No one’s said anything to me directly, but, I do get the feeling that in society you don't discuss it, thrush is a bit, ‘eww,’ and maybe people think you're dirty.

While notions of being ‘dirty’ or ‘gross’ were acknowledged to be linked to thrush, these narratives were also well resisted.

Participants reflected on perceptions that linked recurrent thrush to a lack of femininity. Sasha said that recurrent thrush was ‘*really very uncomfortable and it… sometimes makes you feel you're less of a woman than other women are […] because other women don't go through this*’. The perception that others did not share their experience was another factor that led some participants to feel ‘wrong’ or ‘broken’. Anna felt different from her friends, stating ‘*I was the anomaly in my circle of friends, so I did feel like there was something wrong with me, and I think I carried that for quite a long time that there was something wrong with me*, […] *like my body was wrong*’ and that at one point she felt ‘*defective as a woman*’.

While acknowledging these frameworks, participants also challenged them or presented alternatives. Anna explained that when she was in her 20s, she was expected to see herself a certain way, but that this evolved over time.

I think just being a 20‐year‐old woman, a 20‐year‐old person in a cis‐female body, I don't know how you do it without being full of shame because there’s so much oddness that happens; whereas now like all my friends have pushed babies out of their vaginas and ripped themselves open and had fibroids and had miscarriages and you're like, ‘oh right, these are just like wild vessels’, but I think we don't know [laughs] that when we're 20 and we think they're supposed to be these perfect beautiful things for people to look at, but that’s not what they are. [laughs] So yeah, I would be like you know there’s nothing to be ashamed of, it’s just bodily … it’s literally just bodily fluids and like bacteria.

Anna reflected on the traditional links between bodily fluids, uncleanliness and grossness with unfemininity. However, she was more accepting about the realities of bodily fluids linked in with other biological processes and a shared experience of having a ‘wild vessel’ body.

From another perspective, Teddy acknowledged that while other people may feel like ‘less of a woman’, being nonbinary meant that this did not apply to them.

A lot of people do feel like [recurrent thrush] makes them less of a woman in the way that a lot of cis‐men with erectile dysfunction can feel like they’re less manly for it. It’s that stereotype of like, ‘ah, pristine condition woman’, but I do think that being non‐binary has kind of meant that I didn’t particularly get that. Everything centred on getting some sense of womanhood back, and I was like, ‘that’s not why I want this, I would like to stop being in pain’.

However, while avoiding stigma around aspired femininity, recurrent thrush could be distressing in others facets for gender diverse individuals. KJ and Teddy reflected that having gender dysphoria could also potentially contribute to their vulvovaginal discomfort. Teddy said ‘*If* *you have discomfort with your genitals because of like dysphoria or something, then it might be that you are struggling with other conditions*’.

Further, discomfort due to recurrent thrush could then repeatedly call the genitals into attention and exacerbate gender dysphoria.

### 3.4 Making Compromises About Dis/Comfort

Sometimes the need to manage bodily discomfort came at the expense of the ability for participants to feel comfortable in their gender presentations. This involved people balancing different options that brought discomfort whether it be physical, physiological, or psychological.

One way that people sought to increase physical comfort was through clothing choices, but this could cause discomfort when these choices influenced people's self‐expression and outwards presentations. This included aspects such as clothes rubbing and fabrics or styles causing additional sweating. Emily said ‘*I wasn't able to wear the clothes I wanted at specific times*’. Jody had to give up wearing jeans. Etta said that being nonbinary, the inability to express their gender comfortably through clothing was emotionally difficult. They explained ‘*I can't wear the clothes that I want to wear and express my identity in that way, is a big deal, it's a big deal*’.

I haven't figured out an alternative to dresses and no underwear. I'm trying to find dresses that I feel comfortable in, but I just don't, they're just not my bag. It means I have a particular look that doesn't reflect my inner landscape. That’s really painful.

Etta's description of pain here speaks to the multiple layers of discomfort that people could be managing simultaneously. Although wearing dresses and no underwear could provide physical relief, it led to a ‘really painful’ emotional experience.

In contrast, for KJ, exploring their gender fluid identity helped them find clothing options that provided comfort for both their body and gender.

My identity sort of started to get a bit more … embracing my masc side a little bit more. I would wear men’s cotton pants because I found that they breathed a lot more, and I had a lot less problems with thrush at that point.

Another area of concern raised by participants was physiological discomfort, often tied to medical treatments such as hormonal therapies or contraceptive use, which could bring both relief and complications for recurrent thrush.

Some participants recalled having to make difficult choices between multiple health considerations with a balance of producing and relieving discomforts. Francine began hormone‐replacement therapy to ease the symptoms of menopause, but had to stop this when it was thought to be worsening the recurrent thrush. She explained: ‘*I think the oestrogen can feed the thrush, so I had to stop the HRT patches as well*’.

For Elliott, testosterone‐used to affirm their gender identity as a trans person was a double‐edged sword. Elliot worried that taking testosterone was contributing to recurrent thrush and was unsure what this meant for their gender‐affirming journey.

As soon as I stopped taking T, I stopped having thrush, so then I was just like, ‘oh, maybe I’m just not going to bother because it’s … because I really can’t be bothered with all of like … these like hormonal changes, la‐la‐la’, so I’m sort of … I’m on the fence at the … I mean I’ve got thrush at the moment, which is so annoying, so I’m on the fence, but I don’t really know … I’m in my own sort of journey of like, ‘do I want to keep taking it, do I not?’

This tension of being ‘on the fence’ between what aspects of comfort to prioritise highlighted the compromises around discomfort that came with recurrent thrush.

Similar, considerations around contraception were also described as a double‐bind. Emily saw a doctor about the connection of contraceptive pills and recurrent thrush and said ‘*they took it very seriously, and she wanted to find out the root cause, which I think she's now said is my contraceptive pill which I'm not coming off that*’. Anna described a conversation with her doctor about the link between contraception and recurrent thrush and described his attitude as ‘*Do you want a baby?*’ ‘*No.*’ ‘*Keep taking the pill*’.

Sarah found that the contraceptive coil exacerbated her recurrent thrush:

So me and my partner, we were umming and ahhing it because the coil, if you disregard the thrush, it really suited me, was problem‐free […] eventually it just got to the stage where it’s just like, ‘We don’t need to be on any contraception at all at this stage because we’re not having sex.

Despite the coil being the most comfortable contraceptive option, for Sarah it was not worth the discomfort of recurrent thrush. Zoya said she delayed getting a contraceptive coil altogether, explaining: ‘*I'm already getting [thrush] every other month and I don't want to get in this situation where it's even worse*’.

Lastly, psychological discomfort often stemmed from clashes between participants' self‐identity and societal expectations. The gendered marketing of thrush could cause tension with people's needs to manage their physical suffering. KJ said that they found the labelling around vulvovaginal thrush to be ‘feminine looking’ and Teddy found the marketing ‘very pink’.

Teddy expressed how they had to weigh the discomfort of being perceived as a woman when seeking medication against the discomfort of living with an untreated thrush flare‐up, with the latter often taking priority:

I do think that whenever I go to order medication for thrush, I'm mostly seen as a woman when I go out in public anyway unless I'm in my very … in my little no makeup, I'm dressed like an eboy [masculine‐presenting] kind of look, but that is a time when I'm really like I'm being perceived as a girl right now and I do not like it, but I just kind of have to deal with that because I need the medication, so it’s like I'll tolerate being misgendered for five minutes so that it will stop me from being in pain.

Teddy here acknowledged competing discomforts, one around being misgendered and another from being in physical pain. Etta expressed the emotional pain of not being able to speak freely about the deeply intertwined layers of discomfort, stating that this silence added ‘another layer of inauthenticity’.

## 4 Discussion

This paper explores the ‘mess’ of discomfort caused by recurrent thrush and its attempted management on material, microbial, and gendered levels. This complex process involved repetitively feeling and seeing material mess, self‐monitoring microbiomes, addressing gendered notions of disgust and making compromises about dis/comfort.

### 4.1 Comparisons With Existing Literature

Previous research studies on recurrent thrush and larger discourses of vulval pain have presented gendered notions of ‘failed’ femininity and a loss of ‘womanhood’ (Karasz and Anderson 2003; Adolfsson et al. 2017; Marriott and Thompson 2008; Rees and Arnold 2024). This research tends to overlook, or inadvertently sanitise, the physicality of recurrent vulval discomfort. Previous studies have focused on the emotional and social impacts of recurrent thrush, backgrounding the material realities of this condition, and potentially further reinforcing that aspects of this condition are too ‘disgusting’ or ‘dirty’ to discuss. Discourses about ‘leaky bodies’ have opened up new ways to understand bodily fluids yet have overlooked vulvovaginal discharge, smell and itch as being imbued with meaning and material considerations (Longhurst 2000; Grosz 1994).

Our findings demonstrate that prevalent narratives around recurrent vulvovaginal discomfort reported in past studies miss out on the complexity of gendered and material experiences of embodying a ‘leaky body’. Although participants spoke about dominant narratives around ‘failed femininity’, they did not easily subscribe to these notions nor feel this captured the full depth of their psychological, emotional and relational difficulties. Instead, participants foregrounded the difficulties of managing a materially messy body with a vagina that internally aches and oozes discharge with a vulva that externally itches, splits and scabs.

Participants continuously explored how recurrent thrush was both a genital and gendered problem and the difficulty of managing the sometimes‐competing needs around affirming identity versus eliminating infection. Participants attempted to make sense of living with a condition that did not fit comfortably in their body, nor within existing gendered narratives of vulvovaginal pain. By adding gender‐diverse voices to this study, we gained further insight into the multiple considerations and compromises being made around gendered health and gender affirming care. This opened up new ways to challenge, complicate and reinvent previously taken‐for‐granted links between gender, genitals and infection.

### 4.2 Mess

This paper returns attention to how recurrent thrush materialised in bodily fluids, flows and sensations. Although physical discomfort was often the first sign of recurrent thrush, as recurrence continued, this unease seeped into other areas of people's lives. Recurrent thrush often appeared as a ‘mess’, not only in terms of its material inconvenience but also how it disrupted existing expectations around gender, cleanliness and comportment. Our findings demonstrate how the process of managing the mess of recurrent thrush took place through microbial, material, and gendered imaginaries and realities.

Past theorists have explored notions of dirt, disgust, excess, repulsion, and horror as they relate to bodily fluids, flows, and sensations. Douglas (1966) helps us understand ‘dirt’ as matter out of place. While Douglas often speaks to social and psychological ‘dirt’, recurrent thrush highlights how this ‘dirt’ can materialise into tangible mess. The fluid of vaginal discharge and the physical experience of this spreading, leaking out of or existing within the body were described by participants as illustrating their physical discomfort.

Kristeva (1982) offers insight into abjection as the feeling which can be invoked when the inside/outside boundary is blurred (such as when discharge moves from inside to outside the body). Recurrent thrush exists in the ‘the in‐between, the ambiguous, the composite’ space that Kristeva (1982) describes as it creeps up and reappears at different times. Participants provided insight into what happens when leaking becomes a recurrent, cyclical and excessive process whose ever‐possible presence trickles into their everyday lives.

Leder (1990) deepens our understanding about how order is disrupted when the body and its sensation foreground themselves. Leder's (1990) concept of dys‐appearance describes the return of the body to conscious awareness in moments of disruption, dysfunction or pain. The language of leakage, dys‐appearance and microbial imaginaries offers new analytical tools for understanding chronic and recurrent conditions. Participants described recurrent thrush as itchy, painful and mentally all‐consuming, demonstrating how the condition repeatedly brought the vulva and/or vagina into awareness. The cyclical and recurring nature of thrush meant that the body never fully receded into the background, but remained suspended in an anticipatory state towards the next flare‐up. Just as a leaking body demands attention, sensations, such as itching, burning and irritation, act as phenomenological interruptions—bringing the body into sharp focus and disrupting its taken‐for‐granted state. Exploring leaks not just as boundary violations but as demands for attention allows for a deeper engagement with the affective, temporal and embodied aspects of chronicity, where discomfort oscillates between moments of urgency and invisibility.

**FIGURE 1:**
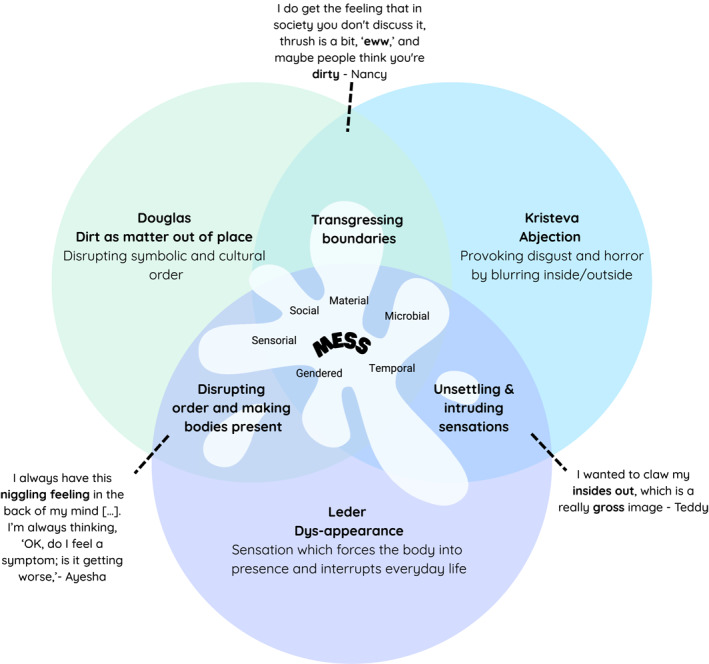
Theoretical underpinning of managing ‘mess’ through the lens of dirt, abjection, and dys‐appearance.

By bringing Douglas, Kristeva, and Leder into conversation, we deepen our understanding of recurrent thrush as a ‘mess’ requiring repeated multi‐layered management. Douglas draws attention to how discharge is constituted as ‘matter out of place’, disrupting the symbolic and cultural order. Kristeva foregrounds how leakage unsettles inside/outside boundaries, provoking abjection, disgust, and horror. Leder highlights how sensations such as itching force the body into focus. These perspectives converge around what might be called *mess*: a social, material, temporal, microbial, gendered and sensorial phenomenon that both transgresses boundaries and demands attention to be ‘cleaned up’. This lens offers insight into understanding recurrently leaky conditions as sites where bodily boundaries, understandings and temporalities are continually unsettled and reconfigured (See Figure 1).

Here ‘mess’ is a physical leak; it is chaos of unexpected timelines, it is disorganisation of identity and referencing how there is not a straightforward solution to unpick. This approach helps to both capture the physical dimensions of this experience but also make room for the multiple layers of material, microbial and gendered dimensions explored in‐depth and the subthemes of temporal, sensorial and social dimensions. This language was chosen out of reclamation, in an attempt to step away from stigmatisation and to focus on the sensorial, not the sensational—illustrating the disruptions recurrent thrush causes and capturing the gravity of a condition that is often dismissed as mundane across policy and clinical agendas.

With recurrent thrush, people had to regulate the ‘mess’ created by their bodies on an enduring, frequent, and repeated basis. The recurrence of thrush moves it from a minor inconvenience requiring short term‐self‐care, and into an all‐consuming occupation with monitoring and managing the body to be kept in ‘balance’. The repetitive and cyclical nature of recurrent thrush also meant that approaches to and understandings of management and self could evolve over time. How people worked to avoid, predict, anticipate, contain, conceal and clean up leaky bodily fluids and sensations could change. This evolution often looked like adding multiple steps to complex management and monitoring routines, and other times it meant shifting perspectives and perceptions of the body and its processes.

### 4.3 Towards an Understanding of Gyn‐Ecology

This paper calls attention to how patients conceptualise and manage the microbiome and recurrent thrush ‘in’ and ‘as’ themselves. This study captures accounts that do not sanitise the experience of recurrent thrush and in doing so deepens our understanding of why and how it matters to patients.

Participants raised questions around how to live in a body that produces a mess, and when it recurrently enters a space of messiness. A range of metaphors from military language to notions of friendship were used by participants to describe their relationships with their microbiomes. Managing recurrent thrush involved not only cleaning up its (visible) material messes but also self‐monitoring the (invisible) microscopic and microbial changes. Exploring thrush through this lens offers an opportunity to deepen our understanding of gyn‐ecological relationships within microbiological imaginaries, particularly as they intersect considerations around the needs of the body and the self.

The vulva has been described as embodying the abject as it transgresses internal/external boundaries as ‘It is elusive, with dark corners and folds, requiring mirrors and a level of mobility to visually self‐examine or self‐explore’ (Rees and Arnold 2024). Rees and Arnold continue that there is shame ‘simply in relation to *having* a vulva, let alone a vulval *disease*’. Further, the appearance of leaking discharge highlights a disjuncture between the anticipated biological function (that the vagina is self‐cleaning and that discharge is a sign of a healthy vaginal microbiome) and material reality (irregular or abnormal discharge). Therefore, a doubling of discomfort and disgust can occur; as vaginal discharge is already stigmatised as dirty then abnormal or ‘infected’ discharge materialises such fears. Further, when such experiences affect gender‐diverse bodies, already steeped in the potential discomfort of dysphoria and antitrans rhetoric of disgust (Vanaman and Chapman 2020; Rydström 2020), these layers were further multiplied.

Discourses of leaking, yeast, and vulvovaginal infection have been closely linked with self‐abjection and disgust (Overend 2011; Braun and Wilkinson 2001). However, participants presented the possibility of moving towards a ‘leaky’ sense of self. Images around ‘wild vessels’, help us reimagine ways to resist often limiting gendered narratives. By embracing the ‘literally just bodily fluids’, people were able to reframe their experiences of leaking and yeast overgrowth as part of a wider experience that did not deserve to be shamed. Yet, despite new narratives emerging around vulvas and leaky bodies (Mowat et al. 2020), stigma still existed as participants worried about how much of their experience was ‘too much’ to describe.

## 5 Implications for Future Research

Our study opens up avenues for considering what a gyn‐ecological approach might look like: one that embraces exploration of the leaks and sensations and critically interrogates gendered narratives. As our paper demonstrates, a more embodied, affective and sensorially attuned framework allows for a richer engagement with how gendered bodies are lived, managed and understood within medical, social and everyday contexts. Other researchers could apply this approach to additional health conditions. What new perspectives appear when we decouple stereotypical gender narratives from the bodies and microbiomes that experience them to explain health experience? What shifts when we turn toward the felt experience of bodily disruption? Centring the sensorial over the symbolic can reveal implicit biases in medical and cultural narratives—such as the pathologisation of certain bodily experiences and the feminisation of certain discomfort(s). This approach also raises questions about whose bodily sensations are deemed appropriate for medical intervention and whose are ignored or normalised through gendered health discourses.

Lastly, we encourage further research into what this approach means for our healthcare management, services and/or training. This is vital to ensure that we produce resources that are responsive and attuned to the material, sensorial and social needs of individuals and communities.

## 6 Conclusion

In this paper, we demonstrated that recurrent thrush has both material realities and gendered meanings that demand repetitive, cyclical, and constant consideration. As thrush recurs, participants often enter into hypervigilant state in monitoring their behaviour, self‐presentation and bodily awareness. Foregrounding the material ‘mess’ of recurrent thrush and how people ‘cleaned up’ leaks, sensations, and spillages, we highlighted the issues that are often side‐lined or sanitised out of research, possibly due to taboos around bodily fluids and vulvovaginal health. In doing so, we re‐centre bodily materiality as a site of lived experience, challenging prevalent narratives that reduce discomfort to psychological distress around ‘failed femininity’. We return attention to the complex material, microbial and gendered discomfort that persistently troubled people with recurrent thrush and place these in conversation with existing theories on dirt, disgust, excess, horror and attention. In doing so, we call for further research exploring dimensions of gyn‐ecology and foregrounding the material and sensorial, rather than only the symbolic, in gender health research.

## Author Contributions

**Tori Ford:** conceptualisation, methodology, investigation, formal analysis, writing – original draft, writing – review and editing, funding acquisition. **Sue Ziebland:** conceptualisation, methodology, formal analysis, writing – review and editing, supervision. **Sarah Tonkin‐Crine:** methodology, formal analysis, writing – review and editing, supervision. **Gail Hayward:** methodology, formal analysis, writing – review and editing, supervision. **Abigail McNiven:** conceptualisation, methodology, formal analysis, writing – review and editing, supervision.

## Funding

This research was funded by an NIHR Doctoral Research Fellowship from the National Institute for Health and Care Research (NIHR) (Grant NIHR302322). The views expressed are those of the authors and not necessarily those of the NIHR or the Department of Health and Social Care.

## Ethics Statement

Ethical approval was granted through the Berkshire research ethics committee (12/SC/0495HTO) for speaking with patients.

## Consent

Informed consent was obtained from all participants in the study. Participants were made aware of the purpose of the research, their right to withdraw, and how their data would be used and stored.

## Conflicts of Interest

The authors declare no conflicts of interest.

## Data Availability

Data from this study is not publicly available, but may be made available upon request.
